# From Experimentation to Optimization: Surface Micro-Texturing for Low-Friction and Durable PTFE–Steel Interfaces Under Full Film Lubrication

**DOI:** 10.3390/polym16243505

**Published:** 2024-12-17

**Authors:** Risheng Long, Jincheng Hou, Yimin Zhang, Qingyu Shang, Chi Ma, Florian Pape, Max Marian

**Affiliations:** 1 Equipment Reliability Institute, Shenyang University of Chemical Technology, Shenyang 110142, China; hjcjc312@163.com (J.H.); zhangyimin@syuct.edu.cn (Y.Z.); sqycan@163.com (Q.S.); 2 Liaoning Provincial Key Laboratory of Efficient Chemical Mixing Technology, Shenyang 110142, China; 3 China-Spain Joint Laboratory on Material Science, Shenyang University of Chemical Technology, Shenyang 110142, China; lg_365@163.com; 4 Institute of Machine Design and Tribology (IMKT), Leibniz University Hannover, An der Universität 1, 30823 Garbsen, Germany; pape@imkt.uni-hannover.de; 5 Department of Mechanical and Metallurgical Engineering, Pontificia Universidad Católica de Chile, Macul 690411, Chile

**Keywords:** surface micro-texturing, PTFE–40# steel tribo-pair, tribological behavior, parameter optimization

## Abstract

To enhance the sliding tribological performance between PTFE and 40#steel (AISI 1040) under full film lubrication conditions, laser surface texturing (LST) technology was employed to prepare micro-dimples on the contact surfaces of 40# steel discs. The Box–Behnken design response surface methodology (BBD-RSM) was applied to optimize the micro-dimple parameters. Coefficients of friction (COFs), wear losses and worn contact surfaces of the PTFE–40# steel tribo-pairs were researched through repeated wear tests, as lubricated with sufficient anti-wear hydraulic oil. The influencing mechanism of micro-dimples on the tribological behavior of tribo-pairs was also discussed. The results proved that micro-dimples can significantly improve the tribological properties of PTFE–40#steel tribo-pairs. The deviation between the final obtained average COF and the prediction by the BBD-RSM regression model was only 0.0023. Following optimization, the average COF of the PTFE–40# steel tribo-pair was reduced by 39.34% compared to the smooth reference. The wear losses of the PTFE ring and 40# steel disc decreased by 91.8% and 30.3%, respectively. This study would offer a valuable reference for the optimal design of key seals used in hydraulic cylinders.

## 1. Introduction

The material, structure and tribological behavior of the main sealing variants (e.g., wiper seal, labyrinth seal, guide-support ring, Glay ring, Y-shaped seal and O-ring) of sealing systems under motion directly affects the performance, service life and reliability of hydraulic cylinders [1]. They also play a crucial role in determining the final production cost and selling price of hydraulic cylinders. Glay rings, composed of an O-ring and a rectangular wear-resistant slider, are commonly used for dynamic sealing between the piston and inner wall, preventing internal leakage within the hydraulic cylinder [2]. The O-ring is typically made of high-elasticity nitrile rubber, while the rectangular slider is usually made of self-lubricating materials, e.g., polytetrafluoroethylene (PTFE), polyoxymethylene (POM) or polyether-ether-ketone (PEEK).During the piston’s reciprocating motion, the large compression stress from the O-ring, combined with the hydraulic pressure from the working chamber, forces the slider to be tightly pressed against the inner wall, forming a small wedge on the low-pressure side and achieving an effective dynamic sealing [2,3]. However, friction and wear between the rectangular slider and the inner wall are inevitable, especially when the piston is misaligned [4] or installed with eccentricity [5,6]. Excessive wear of the rectangular slider can lead to serious internal leakage, shorten the service life of the hydraulic cylinder and increase the energy consumption across the hydraulic system [7,8,9]. Therefore, improving the wear resistance and friction-reducing performance of “rectangular slider–inner wall” tribo-pairs is of great importance. 

Structural optimization is an effective method of reducing the wear of sealing components and prolonging the service life of hydraulic cylinders. For instance, Li et al. proposed a self-compensating variable clearance sealing hydraulic cylinder and developed a load analysis model of the piston lip in the flow field to support design and manufacturing improvements. Three different types of piston structures were also proposed to optimize the sealing performance [10]. Similarly, Hu et al. established a biomimetic sealing ring model, demonstrating that biomimetic sealing rings enhance the sealing performance and durability of hydraulic cylinders [11]. 

Introducing new materials and modifying existing ones is another approach to improving the tribological performance of sealing components. Due to its excellent mechanical properties, self-lubricating capabilities, wear resistance and being chemically inert [12], PTFE is widely used as a sealing material. Xie et al. conducted friction and wear tests on PTFE–GCr15sliding contacts and found that the PTFE debris transferred to the surface of GCr15 steel, forming a solid lubrication transfer film that improved the tribological properties of the PTFE–GCr15 tribo-pair [13]. Li et al. prepared various micro groove textures with different shapes on the surfaces of phosphorus tin bronze discs and tested them against PTFE. They found that the texture pattern reduced the coefficient of friction (COF) due to the formation of a continuous self-lubricating PTFE layer under dry friction, but the wear increased [14]. Other studies also confirmed the transfer of PTFE to different mating materials (e.g., iron [15,16,17], aluminum [15] and bronze [18]), which can reduce friction and lower the wear rates of corresponding mechanical components. Moreover, Kamga et al. used a block-on-ring setup to analyze the formation reasons of PTFE layers on steel rings, finding that PA–PTFE-cb (chemically bonded polyamide matrix and radiation modified PTFE) compounds offered lower COFs and higher wear resistance compared to ordinary polymer materials under ambient conditions [19]. Unal et al. analyzed the wear performance of pure PTFE. They reported that when using various fillers, higher load resulted in lower friction, and fibers like glass fiber could significantly reduce the wear rate [20]. 

Laser surface texturing (LST) technology was first proposed by Hamilton et al. in the 1960s [21]. It offers another effective means to improve the friction and wear performance of friction pairs [22,23,24] or mechanical parts [25] under different lubrication conditions (e.g., dry wear [26], boundary/mixed lubrication [27] and full lubrication [28]), by processing evenly distributed micro-textures (e.g., pits/dimples [27,29,30], grooves [26,31], veins [32,33], multi-scale [34,35] or other functional patterns [36]) with suitable parameters (e.g., shape [27,29,30], size [27,29], depth [30] and area ratio [26]) on contact surfaces (e.g., rolling [26,27], sliding [13] or rolling-sliding [37,38]). Due to its unique features, e.g., high processing accuracy, convenience, relatively fast speed, low cost and environmental friendliness [39,40], researchers have increasingly focused on LST from the perspectives of durability [41], parameter optimization [42] or even increasing friction [43], especially when using 2D materials as lubricants directly [41] or additives [44,45]. 

Regarding the application of LST in seals, Yu et al. demonstrated that LST can enhance the friction performance of the sealing surface and extend the service life of silicon carbide sealing rings under oil lubrication [46]. Razzaque and Faisal conducted a parametric study on a non-contacting mechanical face seals with surface micro-pores of elliptical section through mathematical modeling. They found that better performance in terms of higher clearance and smaller friction torque can be achieved with the proper selection of pore size and pore ratio [47]. Wang et al. investigated the tribological performance of two-phase mechanical face seals with laser textured units on their end faces. Their results revealed that LST shows potential in two-phase mechanical face seals, but the texturing parameters should be carefully considered [48]. Zhang et al. proposed the design of a seal rotor with a micro-surface texture and studied the friction and wear performance of seal pairs under both dry friction and water-lubricated conditions [49]. As for the dynamic sealing of hydraulic cylinders, Chen et al. used biomimetic micro-texturing technology to create textures on the inner surface of hydraulic cylinders, which effectively reduced friction and resulted in a centering force to align the piston [6]. Li et al. found that various shape parameters have different effects on the friction characteristics of textured PTFE–steel pairs, but generally feature the potential to reduce friction [17]. Overall, texturing the inner wall surfaces of hydraulic cylinders can reduce the friction at the sealing interface, improving system efficiency and helping to align the piston.

However, no research has been found on the parameter optimization of micro-textured polymer–steel tribo-pairs. Therefore, to reveal the dynamic tribological behavior between wear-resistant sliders of Glay rings and the inner wall surfaces of hydraulic cylinders, based on previous works [27,29,30,44] and data from Chen et al. [6], an orthogonal experimental method was employed. Micro-dimples with varying diameters, depths and area ratios were designed on the contact surfaces of 40# steel (AISI 1040) discs, which were then tested against PTFE rings lubricated with sufficient anti-wear hydraulic oil. The Box–Behnken design response surface methodology (BBD-RSM) was applied to optimize the micro-dimple parameters, which were then experimentally validated [50]. This work provides valuable insights for the contact surface design of key seals in hydraulic cylinders. 

## 2. Materials and Methods

### 2.1. Materials and Surface Micro-Texturing

**Figure 1:**
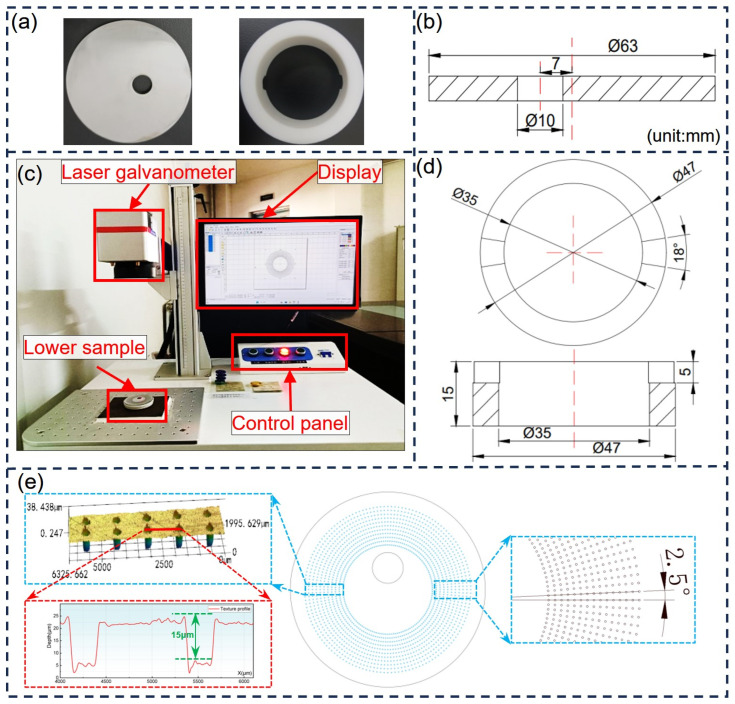
PTFE–40# steel tribo-pair and laser marking system. (**a**) Photos of the steel disc and upper counter rings (PTFE); (**b**) Section view of the lower sample (40# steel disc); (**c**) Photo of laser marking machine; (**d**) Section view of the upper sample (PTFE ring); (**e**) Textured surface of the 40#steel disc before re-polishing and the angle between two adjacent sets of micro-dimples in circumference.

The 40# steel discs were laser-cut and provided by Shandong Kenshi Heavy Industry Machinery Co., Ltd. (Liaocheng, China), with a diameter of 63 mm, a thickness of 5 mm and a surface hardness of HB114.A hole with a diameter of 10 mm and an eccentricity of 7 mm was made to prevent the self-rotating of 40# steel discs during wear tests (see Figure 1a,b). The upper surfaces of the discs were polished with different grades of silicon carbide papers (from 240 to 2000 meshes) and their final surface roughness was about Ra 0.8 μm, measured by a 3D surface profilometer (VK-X1050, Keyence, Osaka, Japan). The counter rings were made of PTFE (see Figure 1a,d), with an inner diameter of 35 mm, an outer diameter of 47 mm and a thickness of 15 mm. Similarly, the lower surfaces of the counter rings were also polished to a surface roughness of Ra 1.1 μm using sandpapers from 240 to 2000 meshes.

A fiber laser marking system (WQ-30W, Wanquan, Shenyang, China) was used to fabricate micro-dimples of varying dimensions (i.e., diameter and depth) on the contact surfaces of the 40# steel discs. Before each laser marking process, the steel disc was ultrasonically cleaned in acetone solution using an ultrasonic cleaner (VGT-1620QTD, Ji’nan, China) for 20 min and then dried using hot air. After the laser marking process, the textured surface of the steel disc was re-polished with sandpaper (from 800 to 2000 meshes) to remove melt bulges (see Figure 1e) along the edges of the micro-dimples [27,29,30]. 

### 2.2. Design of Experiment and the Parameter Optimization Method

Based on previous works [27,29,30,44] and the orthogonal design method, the diameter of dimples (D), the depth of dimples (H) and the area ratios (P) were varied across high, medium and low levels (see Table 1). An experimental design with three factors and three levels was created by Design Expert 10, yielding 17 experimental groups (see Table 2, auto-named by the software). To validate the obtained prediction model, the micro-dimple parameters of the last five groups (from X5-1 to X5-5) were kept identical. A smooth un-structured control group, coded as CT, was introduced as a reference. For those micro-textured groups, there were either 144 or 240 (only T2) uniformly distributed sets of micro-dimples around the circumference (see Figure 1e), with an angle between two adjacent sets in circumference (ABAC) of 2.5° or 1.5°. Each set contained 11–39 micro-dimples, and the total number in a group was determined by its dimple diameter and area ratio. As shown in the figure, the sliding direction of each test was along the circumference of the steel disc, i.e., perpendicular to each set of pits. A3D surface profilometer was also used to check the textured contact surfaces and measure the actual dimple dimensions. The deviations between the designed dimensions of micro-dimples and the actual ones are listed as follows: 200 (±1.8) μm; 250 (+2.4) μm; 300 (±3.2) μm; 5 (+2.2) μm; 15 (+2.3) μm and 25 (+1.4) μm.

In preliminary wear tests, it was found that some PTFE rings exhibited negative mass losses due to the embedding of metal debris, under the conditions of this work. Therefore, the averaged COF was selected as the target parameter and the micro-dimple parameters were optimized using the BBD-RSM quadratic model in Design Expert 10.

Among the data calculated by BBD-RSM, the *F*-value (the ratio of the mean square between groups to the mean square within groups) reflects the relative magnitude of differences between and within groups. A larger *F*-value means a more significant difference between groups. The *p*-value represents the probability of observing the current or more extreme *F*-value under the null hypothesis (i.e., all group means are equal). A smaller *p*-value indicates a greater likelihood that the observed differences between groups are significant. Based on the *p*-values, the significance of each parameter’s effect on the average COF can be evaluated as follows [50]: 

*p*-value < 0.005: very significant (*****); 

0.005 ≤ *p*-value < 0.1: significant (**); 

*p*-value > 0.1: not significant (x). 

According to the obtained prediction model and response surfaces, the optimal micro-dimple parameters were recommended to achieve the lowest average COF for the PTFE–40# steel tribo-pair. Before experimental verification, the micro-dimple parameters should be selected from the recommended and rounded to match the processing precision of the laser marking system and ABACs (1.5° or 2.5°). 

### 2.3. Tribological Test and Characterization

**Figure 2:**
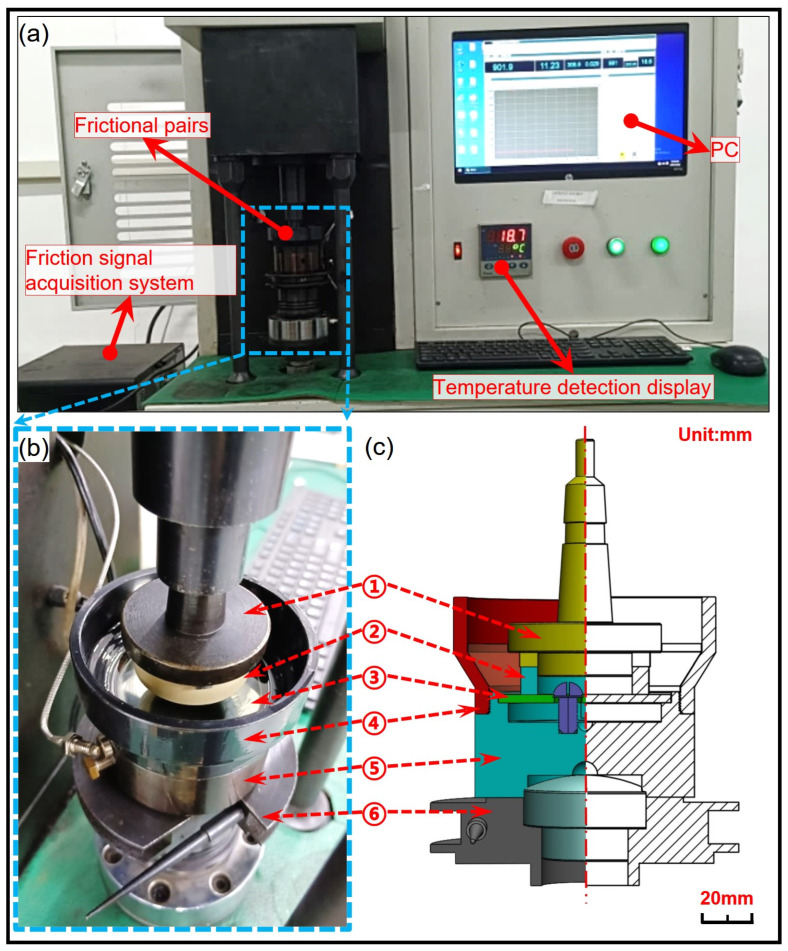
Vertical universal tribological test rig. (**a**) Photo of the MMW-1A vertical universal tribo-meter; (**b**) Photo of the fixtures used; (**c**) Section view of the fixtures: ① upper fixture; ② upper sample (PTFE ring); ③ lower sample (40# steel disc); ④ oil deflector; ⑤ lower fixture; ⑥ loading flange.

As shown in Figure 2, a vertical universal tribological test rig (MMW-1A, Huaxing, Jinan, China) and a ring-on-disc setup were used to study the sliding tribological behavior of the PTFE–40# steel tribo-pairs at room temperature (18 ± 2 °C), as they were lubricated with sufficient anti-wear hydraulic oil (L-HM32, Kunlun, Beijing, China). The thermo-physical parameters of the L-HM32 hydraulic oil are shown in Table 3. 

Based on the typical working conditions (e.g., hydraulic system pressure, ≤25 MPa; movement speed, ≤3 m/s) of key seals (e.g., Glay rings and face seals) applied in hydraulic cylinders, the working parameters of the tribological test rig included: an added vertical load of 1000 N, a rotational speed of 200 rounds per minute (RPM) and a test duration of 2400 s, yielding a sliding distance of approximately 1 km per tribo-pair. 

Prior to each test, 25 mL of hydraulic oil was poured into the lower fixture to fully submerge the 40# steel disc after it was mounted. The COF data were measured directly by the tribological test rig. Due to the large dimensions of the samples in the PTFE–40# steel tribo-pair, the uneven distribution of wear marks, relatively large amount of wear and a large number of micro-dimples with different parameters, mass losses of samples were finally chosen to evaluate their wear resistance rather than volume wear and linear wear. After wear tests, the wear losses were measured using an electronic analytical balance (EX225D, Ohaus, Parsippany, NJ, USA) with an accuracy of 0.1 mg. The final wear loss values were obtained by subtracting the average of nine measurements measured after the wear test from the average of nine measurements measured before the wear test. The worn surface was characterized by a 3D surface profilometer. Note: each group was repeated three times with brand new 40# steel discs to ensure the accuracy of the data obtained and minimize the effects of accidental factors, except for groups from X5-1 to X5-5. These were tested only once for their identical dimple parameters. Three polymer rings were re-polished and reused due to their minimal wear loss in each test. 

## 3. Results and Discussion

### 3.1. COFs

**Figure 3:**
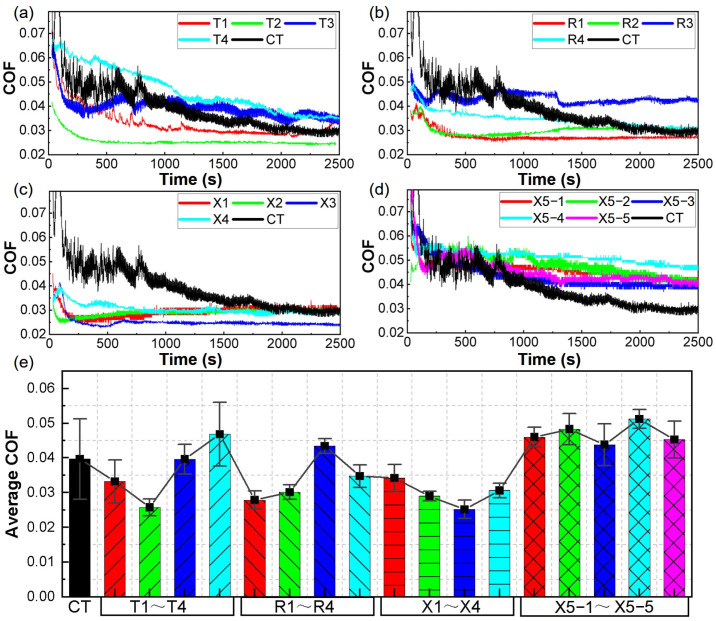
COF data of different groups as the PTFE rings were tested against the 40# steel discs: (**a**) COF curves of T1–T4; (**b**) COF curves of R1–R4; (**c**) COF curves of X1–X4; (**d**) COF curves of X5-1 to X5-5; (**e**) Average COFs of 17 groups.

Figure 3 presents the COF curves for various groups, where PTFE rings were tested against 40# steel discs featuring different micro-dimple parameters. The COF curve of the smooth unstructured group (CT) is also included as a reference. Note: the curves of T1–T4, R1–R4 and X1–X4 represent the average of three repeated tests at each recording time. In the initial stage, all COF curves exhibited high values due to the relatively large static friction coefficients, followed by a sharp decline. Between the 500th s to the 2000th s, the COF curves continued to decrease and gradually became stable in their later periods (from the 2000th s to the 2400th s). The COF curve fluctuation of the smooth group was much greater than the curves of dimple-textured groups, particularly between 0 and the 1000th s. Compared to the smooth reference, the COF curves of the dimple-textured groups were not very high in their earliest time and decreased quite fast, i.e., they reached their stable stages much earlier. Among the groups, T2(D200-P14.9-H15), R1(D300-P6.6-H15) and X3(D250-P6.6-H25) exhibited significantly lower COF curves than the others (see Figure 3a–c). The COF curve of T4 was quite higher than that of T2. This should be attributed to their different area ratios and depth of dimples. 

Specifically, when the diameter of dimples was 200 μm, a higher area ratio was prone to a lower COF curve compared to when the depth of dimples was 15 μm; deeper dimples (with an area ratio of 10.75%) resulted in a higher COF curve (see Figure 3a). When the diameter of dimples was 300 μm, the trends were the opposite (see Figure 3b). Unlike the curves in Figure 3a,b, when the diameter of dimples was 250 μm, four COF curves were quite lower than that of the smooth reference, and the curve of X3 was the lowest (see Figure 3c). Regarding X5-1, X5-2, X5-3, X5-4 and X5–5, as shown in Figure 3d, despite the same dimple parameters, their COF curves still exhibited similar trends and noticeable differences [50]. This should be attributed to the following factors: texture marking quality, re-polished contact surfaces, experimental temperature and minor differences in the operation, etc. Furthermore, the average COFs of most dimple-textured groups were lower than that of smooth reference (see Figure 3e). Therefore, it can be reasonably inferred that micro-dimples have significant friction-reducing effects on the PTFE–40# steel tribo-pairs, and help to reduce the fluctuations of COF curves. The average COFs of X5-1–X5-5 were all higher than that of the smooth group.

### 3.2. Worn Surfaces and Wear Losses

**Figure 4:**
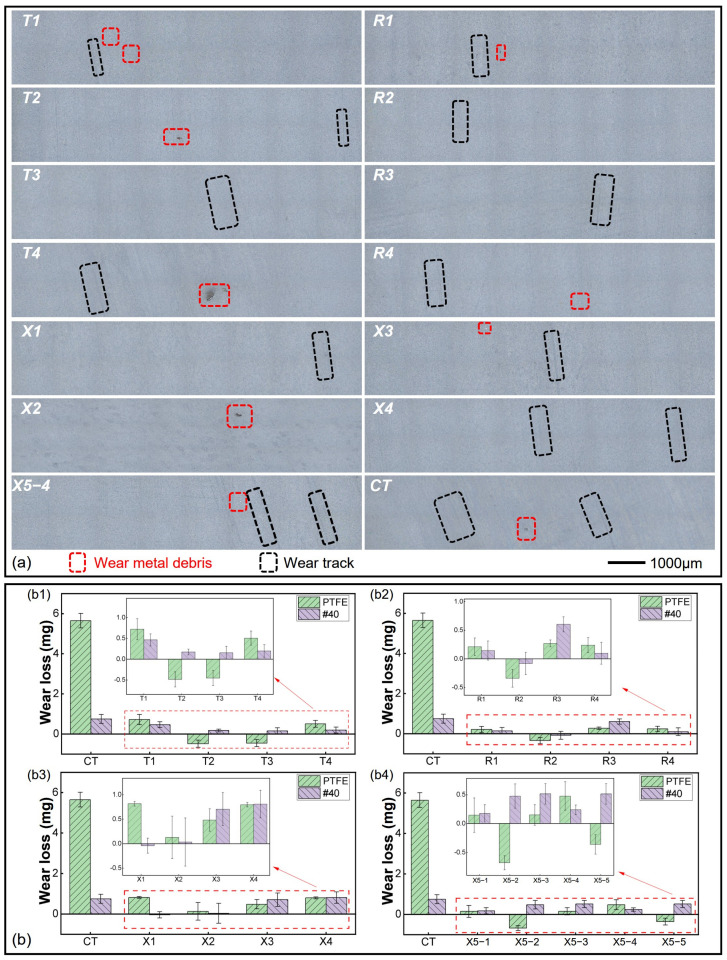
Representative worn surfaces of the PTFE rings and wear losses of different groups after wear tests. (**a**) Worn surfaces of the PTFE rings after ultrasonic cleaning. (**b**) Mass losses of the PTFE rings and 40# steel discs: (**b1**) mass losses of T1–T4; (**b2**) mass losses of R1–R4; (**b3**) mass losses of X1–X4; (**b4**) mass losses of X5-1–X5-5.

The representative worn surfaces of the PTFE rings of different groups after wear tests and ultrasonic cleaning are shown in Figure 4a. The mass losses of the PTFE rings (green) and 40# steel discs (purple) are shown in Figure 4b. There was a large number of small wear marks and obviously embedded metal debris on the worn surface of the PTFE ring for the smooth unstructured group. For the dimple-textured groups, except for a few deeper furrows, the wear marks were less noticeable, but the metal debris embedded in the PTFE rings was still evident. This explains why the masses of some PTFE rings increased after tests and ultrasonic cleaning, e.g., T2, R2, R4, X5-1 and X5-4 in Figure 4(b1–b3). When disregarding the negative data from these limited groups, the wear losses of all dimple-textured groups were much smaller than those of the smooth reference, demonstrating the improved wear resistance of the PTFE–40# steel tribo-pairs enhanced by micro-dimples. Specifically, when the diameter of dimples was 300 μm, i.e., R1–R4, the anti-wear properties of the textured PTFE–40# steel tribo-pairs were notably improved. As the diameter of dimples was small, the smaller area ratio (6.6%) and deeper pit depth (25 μm) were not conducive to improving the anti-wear performance of the tribo-pairs, like T1, T4, X3 and X4. Among the 17 groups, T3, R1, R3 and X5-3 exhibited the outstanding anti-wear properties. In addition, when the area ratio was relatively large (14.9%), the PTFE debris inside the dimples and the PTFE transfer films left on the contact surface were less likely to be completely removed by ultrasonic cleaning, which would slightly increase the masses of the 40# steel discs. This is the reason for the negative wear loss of the 40# steel disc of R2 in Figure 4(b2). Note: given that the accuracy of the electronic analytical balance is only 0.1mg, the small negative wear losses observed in the 40# steel discs of X1 and X5-1, as well as in the PTFE ring of T3, can be disregarded. 

**Figure 5:**
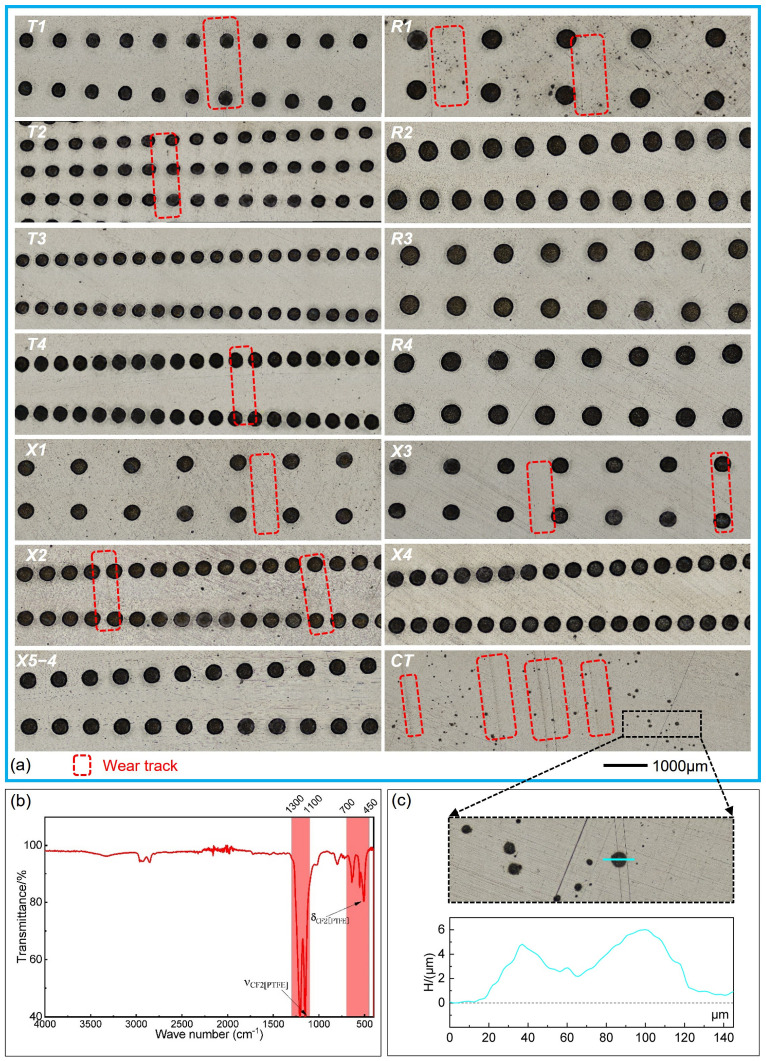
Representative worn surfaces of the 40# steel discs of different groups after wear tests and the FTIR curve of the PTFE transfer film. (**a**) Worn surfaces of the 40# steel discs after ultrasonic cleaning. (**b**) Typical infrared spectral characteristics of the PTFE debris collected from the transfer film on the contact surface of the 40# steel disc. (**c**) Section view of one black spot on the worn surface of the steel disc of the CT group.

**Figure 6:**
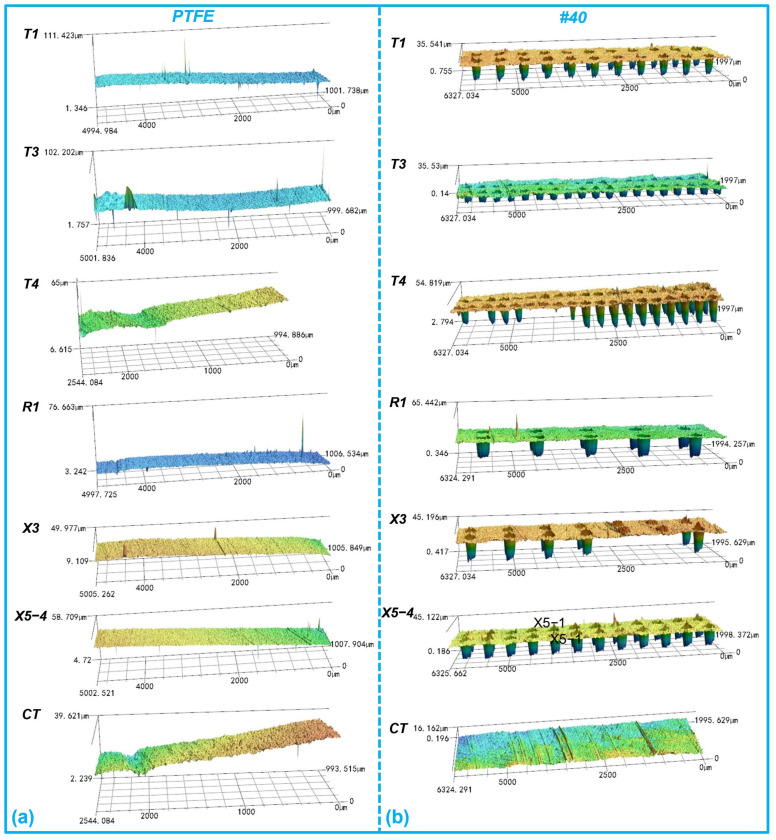
Representative 3D worn morphologies (with an enlargement of 2000% in the height direction) of the PTFE–40# steel tribo-pairs (T1, T3, T4, R1, X3, X5-4) after ultrasonic cleaning. (**a**) PTFE rings; (**b**) 40# steel discs.

The representative worn surfaces of the 40# steel discs after ultrasonic cleaning are shown in Figure 5a. Similarly, there were more prominent wear marks on the contact surface of the smooth 40# steel disc, and the wear marks on the dimple-textured steel discs were less visible, as confirmed by the 3D worn surfaces in Figure 6. Different colors on the surface indicated different depths. The colors of different groups were not comparable and determined by the height difference at different positions in the image. 

It should be noted that in groups like T4 and X3, due to the large depth of dimples, some dimples on the 40# steel discs were blocked by wear debris and could not be completely removed after ultrasonic cleaning. This suggests that the actual wear losses of these steel discs might be slightly higher than the calculated values. This is because a large amount of PTFE debris was generated during the testing process, and locally distributed discontinuous transfer films formed on the contact surface under full film lubrication (both in smooth and dimple-textured groups) [13,14,19]. When the diameter of dimples and area ratios were relatively smaller, the PTFE debris inside the dimples was repeatedly squeezed and gradually became compacted during the formation of the transfer film. This was especially apparent when the depth of the dimples was deeper (25 μm), i.e., T4 and X3. This eventually led to the blocking of some dimples by accumulated PTFE, which could not be effectively removed under ultrasonic cleaning (see Figure 6b). The transfer film and wear debris were collected from the steel disc and analyzed by Fourier transform infrared spectroscopy (FTIR, IS10, Thermo Fisher Scientific, Waltham, MA, USA). The typical infrared absorption peaks of the main functional groups of PTFE can be found in the range of 1400~400 cm^−1^ in Figure 5b (red line) [51]. 

Additionally, there were many small black spots on the worn surface of the 40# steel disc of smooth group (see Figure 5a). The height of these spots were a little higher than the entire surface (about 4 μm, see the cyan line in Figure 5c). This should be attributed to cavitation caused by the collapse of micro air-bubbles during the continuous compression and rotation of PTFE ring under lubrication with sufficient anti-wear hydraulic oil [52]. After ultrasonic cleaning and drying with hot air, the cavitation regions quickly rusted, then turned black and protruded from the entire contact surface, without the protection of the oxide film. This phenomenon was greatly improved in those micro-dimple textured groups, highlighting the effectiveness of micro-dimples in eliminating and reducing cavitation on the contact surfaces of the PTFE–40# steel tribo-pairs. Specifically, severe cavitation was only observed on the worn surface of the 40# steel disc of R1. The steel discs of X1 and X2 showed fewer cavitation-induced black spots on the worn surfaces. The cavitation in other groups was not obvious, especially when the area ratio was relatively high and the depth of the dimples was large. The reason for the serious cavitation on the worn surface of R1 should be attributed to its larger dimple diameter (300 μm) and smaller area ratio (6.6%). In this case, the distance between adjacent pits was very far and the inhibitory effect of micro-dimples on cavitation was not significant. 

Due to the fact that the experimental grouping was designed based on the orthogonal experimental design method, it was very hard to analyze the effects of the three factors on the friction performance of the PTFE–40# steel tribo-pairs comprehensively. So, the BBD-RSM was used to evaluate the significance of the influence of these three factors (D, H and P) on the tribological performance of the tribo-pairs. 

## 4. Parameter Optimization of Micro-Dimples by BBD-RSM

After wear tests, the average COFs of the 17 groups in Figure 3e were input for the BBD-RSM analysis (see Table 4). Note: each average COF in the table is the average of the entire COF curve of the corresponding group in Figure 3. The obtained variances from the derived BBD response surface quadratic model are listed in Table 5. The *p*-value of the proposed model was only 0.0004, indicating that the accuracy of this model was very high. The *p*-value of *P*^2^ was even less than 0.0001, implying that the square of the area ratios had a very significant effect on the average COFs. Other factors, such as *DH*, *PH*, *D*^2^ and *H*^2^, also had significant impacts on the target COFs, for their lower *p*-values (less than 0.1). 

In addition, the determination coefficient (*R*^2^) and correction determination coefficient (*R*^2^_Adj_) were both close to 1. The difference between the predicted correction coefficient (*R*^2^_Pre_) and correction determination coefficient (*R*^2^_Adj_) were also less than 0.2. This suggested that the proposed model could reflect the real situation. The signal-to-noise ratio (*PA*) exceeded 4, implying that the model is reliable and can be used for the parameter optimization [50]. 

The final regression equation of the prediction model can be written as the following:

(1) $$\begin{array}{l} \mu =-0.11448+6.00802\times 10^{-4}D+0.014135P+1.98155\times 10^{-3}H+1.17362\times 10^{-5}DP-7.94309\times 10^{-6}DH\\ +6.40013\times 10^{-5}PH-1.26193\times 10^{-6}D^{2}-8.45437\times 10^{-4}P^{2}-2.64845\times 10^{-5}H^{2} \end{array}$$

**Figure 7:**
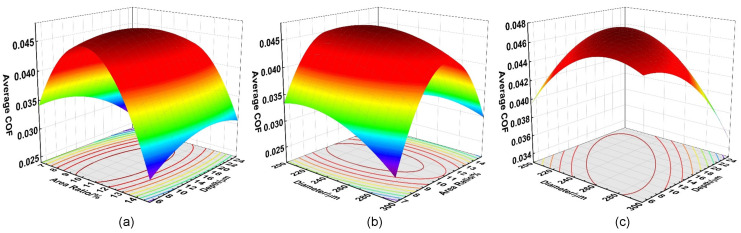
Response surfaces of the interaction among three factors (*D*, *P*, *H*) on the average COFs of the PTFE–40# steel tribo-pairs. (**a**) Response surface among average COF, *P* and *H*; (**b**) Response surface among average COF, *D* and *P*; (**c**) Response surface among average COF, *D* and *H*.

The response surfaces of the average COF as a function of the three micro-texture parameters (*D*, *P*, *H*) are shown in Figure 7. The range of the two horizontal axes of a response surface coincided with their factor level (Table 1).When the value of one horizontal axis is fixed, the response surface displays a downward-opening parabola along the vertical axis (average COF as the dependent variable, with the other horizontal axis as the independent variables). In this case, the more significant the influence of a horizontal axis, the smaller the opening of the parabola. As a result, the entire response surface becomes much sharper along the vertical axis. Additionally, blue in Figure 7 represents the area with the greatest impact significance, followed by green and red. 

So, based on the degree of protrusion of the response surfaces, the influence of micro-dimple parameters on the average COF follows the order: *P* > *D* > *H*, which is consistent with the *p*-values of *P*^2^, *D*^2^ and *H*^2^. To maintain the same angle between adjacent sets of micro-dimples (see Figure 1e, i.e., 1.5°/2.5°), the following parameters were selected from the combinations recommended by BBD-RSM: *D* =297.37 μm, *P* =6.91% and *H* = 22.35 μm, with a target average COF of 0.0222. Prior to laser marking, those parameters were rounded to *D* = 300 μm, *P* = 6.6% and *H* = 20 μm, based on the precision and performance of the laser marking system. The target average COF was 0.0217 after this adjustment. 

## 5. Experimental Validation

**Figure 8:**
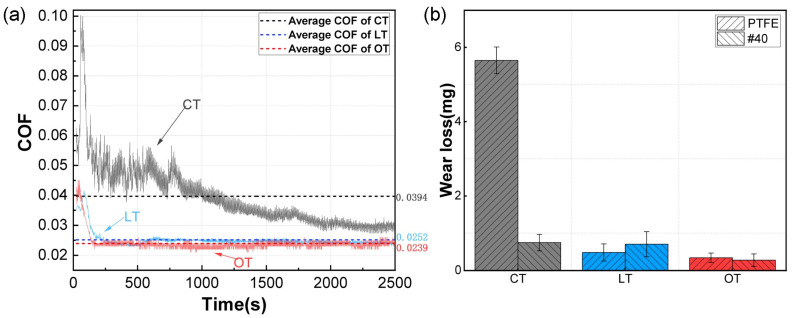
COF curves and wear losses of three groups (OT, LT and CT). (**a**) COF curves and average COF lines of OT, LT and CT; (**b**) Wear losses of OT, LT and CT.

To validate the tribological performance after BBD-RSM optimization, the micro-dimples with the rounded parameters were manufactured on the contact surfaces of 40# steel discs, which were then tested against the PTFE rings under the same conditions: a vertical load of 1000 N, a rotational speed of 200 RPM, a test duration of 2400 s and lubricated with sufficient hydraulic oil. The COF curve of the optimized dimple-textured group (this group was labeled as OT), the lowest COF curve among 17 dimple-textured groups in Figure 3 (this group, i.e., X3, was coded as LT) and the reference curve of the smooth group (CT) are compared in Figure 8a. Note: the OT group was tested three times using three brand new 40# steel discs, and each COF curve shown was the average of three repeated tests at each recorded time. All three COF curves were also quite high at the start and then sharply decreased. In the end, they became stable in their later periods (from the 500th s to the 2000th s). Compared with the curves of LT and OT, the COF curve of the smooth reference fluctuated more dramatically, especially during the early period (from the 0th s to 800th s), taking significantly longer to stabilize. Therefore, it can be reasonably inferred that micro-dimples can significantly reduce the COF fluctuations in PTFE–40# steel tribo-pairs as lubricated with sufficient hydraulic oil, and greatly accelerate their time to be stabilized. This improvement has significant implications for enhancing the dynamic performance and service life of hydraulic cylinders. Compared to LT and CT, the average COF of OT was reduced by 5.2% and 39.3%, respectively. The deviation between the experimentally obtained average COF (i.e., 0.024) and the adjusted target average COF (0.0217) predicted by BBD-RSM was only 0.0023, demonstrating the effectiveness of the BBD-RSM regression model. 

The wear losses of three groups are shown in Figure 8b. Obviously, the wear losses were the highest in the CT group, for both the PTFE ring and the 40# steel disc, followed by LT. The OT group exhibited the lowest mass losses for both parts. Compared to the CT group, the wear losses of the PTFE ring and 40# steel disc in the OT group were decreased by 91.8% and 30.3%, respectively. Similarly, the wear losses of the PTFE ring and 40# steel disc in the OT group also decreased by 70.7% and25.9% compared with the results of LT, respectively. This indicates that the optimization parameters of micro-dimples can also significantly enhance the wear resistance of PTFE–40# steel tribo-pairs. 

**Figure 9:**
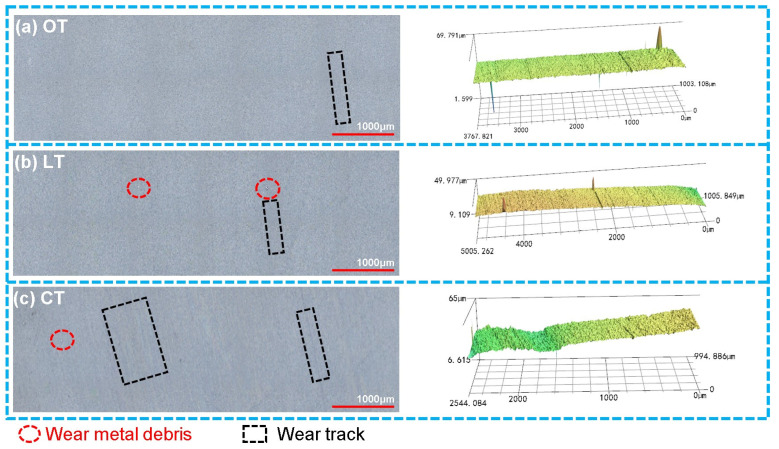
Representative worn surfaces and 3D morphologies of the PTFE rings of OT, LT and CT after ultrasonic cleaning. (**a**) OT; (**b**) LT; (**c**) CT.

**Figure 10:**
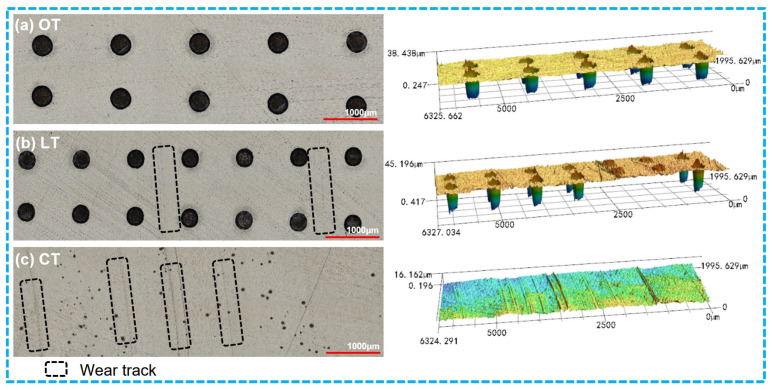
Representative worn surfaces and 3D morphologies of the 40# steel discs of OT, LT and CT after ultrasonic cleaning. (**a**) OT; (**b**) LT; (**c**) CT.

The representative worn surfaces and 3D morphologies of the PTFE rings from the three groups (OT, LT and CT) are shown in Figure 9. Similarly, different colors on the surface indicated different depths. The colors of different groups were not comparable and determined by the height difference at different positions in the image. Unlike the many wear marks on the worn surface of CT and the slight wear marks on the worn surface of LT, there were only very few wear marks on the worn surface of OT, with no visible embedded metal debris (see Figure 9a). Regarding the 40# steel discs, there were only very slight wear marks on the worn surface of the steel disc of OT (see Figure 10), confirming that the optimized dimple parameters can effectively enhance the wear resistance of PTFE–40# steel tribo-pairs. 

## 6. Influence Mechanism of the Micro-Dimples on the Tribological Performance of PTFE–40# Steel Tribo-Pair

**Figure 11:**
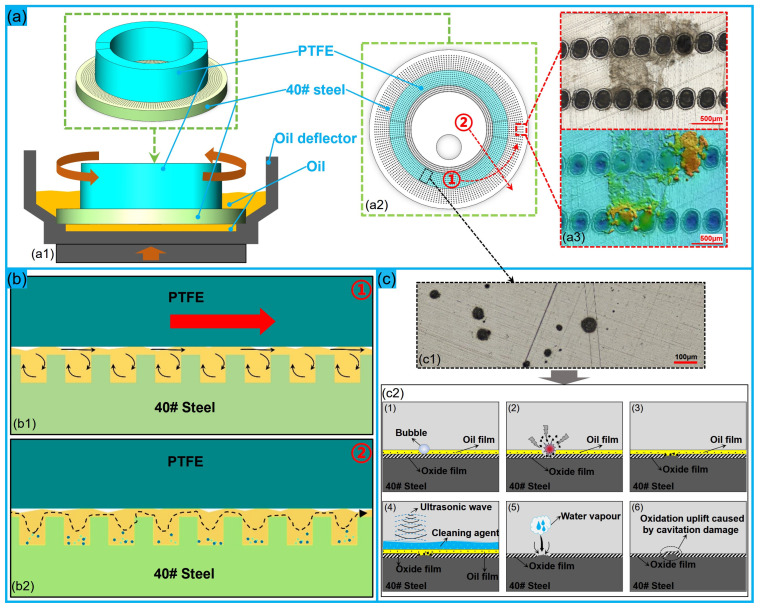
Influence mechanism of micro-dimples on the tribological performance of the PTFE–40# steel tribo-pair as lubricated with sufficient anti-wear hydraulic oil. (**a**) PTFE–40# steel tribo-pair and transfer film: (**a1**) section view of PTFE–40# steel tribo-pair; (**a2**) top view of PTFE–40# steel tribo-pair; (**a3**) transfer film left on the surface. (**b**) Micro-eddies in micro-dimples and the debris migration: (**b1**) influence of micro-eddies on the load-carrying capacity along the circumference; (**b2**) migration of wear debris along the radius direction. (**c**) Cavitation phenomenon and its formation mechanism: (**c1**) image of black spots; (**c2**) formation mechanism of cavitation.

The main functions of the micro-dimples include entrapment of wear debris, lubricant storage and the enhancement of load-carrying capacity. In this work, all wear tests were conducted under full film lubrication, i.e., the steel discs were completely immersed in the anti-wear hydraulic oil throughout the whole procedure (see Figure 11a). Therefore, in this case, the influencing mechanism by which micro-dimples influence the friction and wear performance of PTFE–40 # steel tribo-pairs can be summarized as follows:

(1) As the PTFE ring begins to rotate, owing to the sufficient amount of hydraulic oil, the micro-dimples fill with hydraulic oil and a full hydraulic oil film lubrication is gradually generated between the contact surfaces of the PTFE–40# steel tribo-pair. Compared to the smooth reference, the hydraulic oil in micro-dimples can be squeezed out and migrate radially under the action of centrifugal force as the PTFE ring rotates [53], maintaining a continuous hydraulic lubricating oil film in the contact zone. By acting as numerous “micro-hydrodynamic bearings” (see Figure 11(b1)) through generating a hydrodynamic pressure build up between oil-lubricated parallel sliding surfaces, the micro-dimples can significantly slow down the lubricant migration in the contact regions and increase the load-carrying capacity of the oil film. This is the reason for the significantly improved tribological performance of the dimple-textured groups [21,27].
(2) With the fast rotation of the PTFE ring, a large amount of polymer particles and a small amount of metal debris is gradually generated. Micro-dimples can effectively entrap those particles/debris carried by oil and store them during the continuous radial migration of the lubricant (see Figure 11(b2)), reducing the amount of rigid metal debris remaining in the contact regions [52], especially as the tribo-pair is tested under full film lubrication or elastohydrodynamic lubrication (EHL). Due to the low surface hardness of the PTFE, there is an unavoidable but small amount of rigid debris embedded into the contact zone of the PTFE ring. This is the reason for the negative wear losses of PTFE rings in some groups (see Figure 4b) and why the COF was chosen as the target parameter in BBD-RSM.
(3) During the high-speed rotation of the PTFE ring, PTFE particles can also be transferred to the steel disc to form a transfer film (see Figure 11(a3)) [13,14,19], due to the local frictional heat and continuous pressure from the PTFE ring. This film helps to reduce the COF and wear losses of the tribo-pair, though it is weakly bonded to the steel surface due to the low surface energy of PTFE. Correspondingly, the film (with varying sizes) is discontinuously distributed on the contact surface of the 40# steel disc, and can be easily removed by ultrasonic cleaning. However, owing to the “self-sealing” effect of micro-textures [27,30], the continuous pressure of the PTFE ring, and the depth of the micro-dimples, accumulated PTFE particles can block the micro-dimples and cannot be completely removed. This makes the actual wear loss of the steel disc slightly higher than that calculated in Figure 4b.
(4) The diameter of the micro-dimples and the area ratios determine the number and spacing of dimples on the textured surface, thus reducing the real contact area of the tribo-pair. A larger diameter of dimples and smaller area ratio correspond to a larger spacing between adjacent dimples, which is beneficial for maintaining good micro-eddies to improve the load-bearing capacity of the contact surface. Micro-dimples also help to minimize the cavitation phenomenon on the 40# steel discs (see Figure 11(c1,c2)), especially when the area ratio is relatively large and the dimples are not very deep. When the diameter of the dimples and the area ratio is constant, the depth of the dimples mainly affects the micro-eddies and “self-sealing effect”. A small dimple depth is not conducive to the formation of micro-eddies and the collection of debris, while a large dimple depth is also not ideal due to the “self-sealing effect”, in this case. Additionally, the laser-induced phase transitions, such as melting followed by rapid solidification or the “U-shaped thin-wall embedded units” [54], may also modify the intrinsic material structure, which could increase the hardness of a near-surface layer to improve the wear resistance of textured surfaces [27].

Therefore, unlike the results under dry wear conditions [26,55,56], the final tribological behavior of a dimple-textured PTFE–40# steel tribo-pair is mainly determined by the following factors: the area ratio of the textured contact surface, dimensions of micro-dimples (i.e., diameter and depth), surface contact stresses, transfer film properties (i.e., thickness and distribution), “U-shaped thin-wall strengthening effect” of micro-dimples, thermo-physical properties of the hydraulic oil, load, rotating speed and so on. 

## 7. Conclusions

Based on the orthogonal experimental design method, micro-dimples with different parameters (i.e., diameter, depth and area ratios) were processed on 40# steel discs by laser texturing and tested against PTFE rings lubricated with sufficient anti-wear hydraulic oil. Then, the dimple parameters were optimized using BBD-RSM and validated experimentally. Based on the results obtained, the following conclusions can be drawn:

(1) Micro-dimples can significantly reduce the COF fluctuations of the PTFE–40# steel tribo-pairs under full film lubrication and reduce the time to be stable. Most average COFs and all wear losses of dimple-textured groups were lower than those of the smooth unstructured reference, indicating the friction-reducing and anti-wear performance of the micro-dimples in this study. The embedded metal debris in PTFE rings and the PTFE debris stored in deep dimples accounted for the negative mass losses of some groups, e.g., X1, X2 and X3.
(2) Compared with the black spots on the worn surface of the 40# steel disc of the smooth reference, it is expected that micro-dimples can effectively eliminate and weaken the cavitation which takes place on the textured 40# steel discs, especially if the area ratio is relatively large and the dimples are not very deep.
(3) Based on the analysis of BBD-RSM and the obtained *p*-values, the influence of dimple parameters on average COFs followed the order: *P* > *D* > *H*. Compared to the LT and CT groups, the average COF of the OT group was reduced by 5.2% and 39.3%, respectively. The deviation between the experimentally obtained average COF and the adjusted target average COF predicted by BBD-RSM was only 0.0023. Compared with the wear losses of the LT group, the mass losses of the PTFE ring and 40# steel disc in the OT group decreased by 70.7% and25.9%, respectively. This indicates that the micro-dimple parameters after optimization can significantly improve the tribological behavior of the PTFE–40# steel tribo-pairs.

These findings can provide valuable insights for the optimization design of the main seals used in hydraulic cylinders.

## Figures and Tables

**Table 1:** Orthogonal factor-level table.

Level	Factor		
	Diameter of Dimples, D (μm)	Depth of Dimples, H (μm)	Area Ratio, P (%)
−1	200	5	6.6
0	250	15	10.75
1	300	25	14.9

**Table 2:** Experimental scheme created by Design Expert10 and their ABACs.

Group ID	Diameter of Dimples D (μm)	Area Ratio P (%)	Depth of Dimples H (μm)	ABAC * (°)
T1	200	6.6	15	2.5
T2	200	14.9	15	1.5
T3	200	10.75	5	2.5
T4	200	10.75	25	2.5
R1	300	6.6	15	2.5
R2	300	14.9	15	2.5
R3	300	10.75	5	2.5
R4	300	10.75	25	2.5
X1	250	6.6	5	2.5
X2	250	14.9	5	2.5
X3	250	6.6	25	2.5
X4	250	14.9	25	2.5
X5-1	250	10.75	15	2.5
X5-2	250	10.75	15	2.5
X5-3	250	10.75	15	2.5
X5-4	250	10.75	15	2.5
X5-5	250	10.75	15	2.5

* ABAC: The angle between two adjacent sets of micro-dimples in circumference.

**Table 3:** Thermo-physical properties of the L-HM32 anti-wear hydraulic oil.

Physical Property	Value
Density (g/cm^3^@20 °C)	0.85
Viscosity (mm^2^/s@40 °C)	30.69
Flash point (°C)	220
Pour point (°C)	−15
Condensation point (°C)	−130

**Table 4:** The average COFs of 17 groups in Figure 3e input for the BBD-RSM analysis after wear tests.

Group Name	Average COF
T1	0.0331
T2	0.0257
T3	0.0396
T4	0.0468
R1	0.0278
R2	0.0301
R3	0.0434
R4	0.0347
X1	0.0342
X2	0.0290
X3	0.0252
X4	0.0306
X5-1	0.0460
X5-2	0.0483
X5-3	0.0438
X5-4	0.0512
X5-5	0.0453
CT	0.0397

**Table 5:** Variance analysis of the BBD response surface quadratic model.

Source	Sum of Squares	Mean Square	*F*-Value	*p*-Value	Significance
Model	1.152 × 10^−3^	1.280 × 10^−4^	18.56	0.0004	*****
*D*	1.071 × 10^−5^	1.071 × 10^−5^	1.55	0.2527	x
*P*	3.023 × 10^−6^	3.023 × 10^−6^	0.44	0.5291	x
*H*	9.812 × 10^−6^	9.812 × 10^−6^	1.42	0.2719	x
*DP*	2.372 × 10^−5^	2.372 × 10^−5^	3.44	0.1061	x
*DH*	6.309 × 10^−5^	6.309 × 10^−5^	9.15	0.0193	**
*PH*	2.822 × 10^−5^	2.822 × 10^−5^	4.09	0.0828	**
*D* ^2^	4.191 × 10^−5^	4.191 × 10^−5^	6.08	0.0432	**
*P* ^2^	8.927 × 10^−4^	8.927 × 10^−4^	129.41	<0.0001	*****
*H* ^2^	2.953 × 10^−5^	2.953 × 10^−5^	4.28	0.0773	**
Residual	4.829 × 10^−5^	6.898 × 10^−6^			
Lack of Fit	1.472 × 10^−5^	4.905 × 10^−6^	0.58	0.6563	x
Pure Error	3.357 × 10^−5^	8.393 × 10^−6^			
Cor Total	1.200 × 10^−3^				
*R*^2^ = 0.9598	*R*^2^_Adj_ = 0.9081	*R*^2^_Pre_ = 0.7602	*PA* = 10.273		

Notes: ***** very significant (*p* < 0.005); ** significant (*p* < 0.1); x notsignificant (*p* > 0.1).

## Data Availability

The original data used to support the findings of this study are available from the corresponding author upon request.
